# Establishment of an efficient *Agrobacterium rhizogenes*-mediated hairy root transformation system for functional analysis in tomato

**DOI:** 10.3389/fpls.2026.1968539

**Published:** 2026-10-14

**Authors:** Siyi Zhang, Longzhu Huang, Junjie Liu, Zhimin Yin, Ziwei Li, Yuanhui Song, Qiuling Zhang, Tengbo Huang

**Affiliations:** 1 Guangdong Provincial Key Laboratory for Plant Epigenetics, College of Life Sciences and Oceanography, Shenzhen University, Shenzhen, Guangdong, China; 2 College of Physics and Optoelectronic Engineering, Shenzhen University, Shenzhen, Guangdong, China; 3 Ganzhou Sub-Center of National Vegetable Quality Standards Center, Ganzhou, Jiangxi, China

**Keywords:** Agrobacterium rhizogenes, functional studies, hairy root, MicroTom, SlMYB28

## Abstract

Tomato is an important model for functional genomics in Solanaceae, and elucidating its gene functions provides valuable insights into conserved genetic regulatory networks across dicotyledonous plants. Hairy root transformation is a key technology for functional studies in tomato. However, systematic optimization of its parameters remains limited. Here, we established an efficient *Agrobacterium rhizogenes*-mediated hairy root transformation system for tomato cv. Micro-Tom. Using the RUBY visual reporter gene, we systematically evaluated multiple parameters, including *A. rhizogenes* strain, bacterial optical density (OD_600_), inoculation duration, acetosyringone (AS) concentration, and the type and concentration of antibiotics for *Agrobacterium* eradication. ArQual was identified as the most suitable strain. Optimization revealed that an OD_600_ of 0.7, inoculation duration of 10 min, and AS concentration of 200 μM, these optimized conditions yielded the highest transformation efficiency of 66.01%. To validate the utility of this system, we first demonstrated that overexpression of *SlMYB28* promotes anthocyanin accumulation in stable transgenic lines. We then applied the optimized hairy root transformation system to functionally characterize *SlMYB28*, achieving 59.33% transformation efficiency and distinct purple pigmentation in transgenic roots. These results support the positive regulatory role of *SlMYB28* in anthocyanin biosynthesis and demonstrate the utility of this system for rapid visual validation of stress-responsive genes. This rapid, efficient, and visually tractable transformation platform provides a useful tool for rapid functional screening and preliminary characterization in tomato.

## 1 Introduction

Tomato (*Solanum lycopersicum*) is one of the most economically and nutritionally important vegetable crops worldwide and serves as a model system for studying fruit development, disease resistance, secondary metabolism, and hormone signaling (Klee and Giovannoni, 2011;Liu et al., 2022; Bai et al., 2025; Becze et al., 2025; Wang and de Maagd, 2025). The Micro-Tom cultivar is widely recognized for functional genomics due to its compact size, short life cycle, and well-characterized genetic background (Sun et al., 2006; Gasparini et al., 2025). Advances in functional genomics and molecular breeding have heightened the demand for efficient and stable genetic transformation systems. Although stable genetic transformation of tomato has been achieved, primarily through *Agrobacterium tumefaciens*-mediated transformation of cotyledon or hypocotyl explants, this approach faces several limitations, including time-consuming procedures, low efficiency, and strong genotype dependence (Nonaka et al., 2019; Sakthivel et al., 2025). By contrast, *Agrobacterium rhizogenes*-mediated hairy root transformation system offers operational simplicity, rapid turnaround, and stable transgene expression, and has been widely applied in gene function analysis, particularly in roots (Ron et al., 2014; Biswas et al., 2023; Niazian et al., 2023; Zhang et al., 2025). This system facilitates rapid functional analysis of genes, advancing our understanding of genetic regulatory networks in tomato, and offering valuable insights for functional gene studies in related crops of economic importance.

*A. rhizogenes*, a Gram-negative bacterium with a broad host range, harbors T-DNA on its *Ri* plasmid. Since the 1974 discovery that the *Ri* plasmids induce hairy root formation, this system has evolved into a versatile platform for genetic transformation and plant-microorganism interaction studies (Gantait and Mukherjee, 2021; Shi et al., 2021; Loyola-Vargas et al., 2024). Hairy roots provide numerous advantages for genomic research, including efficient transformation, high genetic stability, rapid growth under simple culture conditions, convenience for genome editing operations, and useful representation of root biology (Demchenko and Pawlowski, 2021; Nguyen et al., 2022; Loyola-Vargas et al., 2024). The technology has been established in diverse species, including apple (*Malus domestica*), blueberry (*Vaccinium* *corymbosum*), litchi (*Litchi chinensis*), and loosestrife (*Lythrum salicaria*) (Qin et al., 2021; Ebrahimi et al., 2023; Liu et al., 2024; Zhong et al., 2025; Kumam et al., 2026). However, the hairy root induction efficiency varies substantially across plant species and *A. rhizogenes* strains. For instance, loosestrife achieved a 65% induction rate with strain A4 using excised shoots as explants (Ebrahimi et al., 2023), whereas in *Physalis peruviana*, strains R1601, C58C1, and ArA4 achieved 57.78%, 65.14%, and 72.31%, respectively, while strain K599 completely failed (Zhong et al., 2025). Such strain- and species-dependent variation necessitates systematic optimization of key parameters, including bacterial strain, bacterial suspension density (OD_600_), inoculation duration, acetosyringone (AS) concentration, and antibiotic selection, to maximize transformation efficiency (Thwe et al., 2016; Sathasivam et al., 2022; Niazian et al., 2023; Pan et al., 2025). Accordingly, strain R1601 proved optimal for *Ocimum basilicum* (Sathasivam et al., 2022), R1000 for soybean (*Glycine max*) (Niazian et al., 2023), and K599 for passion fruit (*Passiflora edulis*) (Pan et al., 2025). In *Solanum lycopersicum* cv. Ailsa Craig, efficiencies of 40-60% have been reported with various strains (Bian et al., 2022), yet systematic optimization of transformation parameters for *Solanum lycopersicum* cv. Micro-Tom remains to be established.

Recent advances in visual reporter genes have greatly facilitated the detection of transformants. RUBY is an artificial operon encoding all the enzymes required for betalain biosynthesis, and its effectiveness as a visible reporter gene has been validated in both monocots and dicots (He et al., 2020; Niazian et al., 2023). Unlike conventional reporters such as GUS, GFP, and luciferase, which rely on exogenous substrates, specialized instrumentation, or destructive detection methods, RUBY enables real-time, non-invasive monitoring of transgene expression throughout hairy root development (Yu et al., 2023). This reporter has been successfully deployed in soybean, *Plukenetia volubilis*, and *Nicotiana benthamiana*, significantly streamlining the screening process for transgenic hairy roots and reducing the time and cost of gene functional analysis (Yu et al., 2023; Niazian et al., 2023).

Phosphorus (P) is an essential macronutrient, and its bioavailability is often limited by fixation into insoluble forms in most soils (López-Arredondo et al., 2014; Zhang et al., 2024; Singh et al., 2026). Plants respond to phosphate deficiency through a range of adaptive responses, including the accumulation of anthocyanins (López-Arredondo et al., 2014; Zhang et al., 2019). Anthocyanins are water-soluble flavonoid pigments that accumulate in vacuoles under various environmental stresses, including phosphate deficiency, drought, cold, and high light intensity (Zhang et al., 2019; Lin et al., 2025). As antioxidant metabolites, anthocyanins protect plant cells from oxidative damage and contribute to stress tolerance, making them valuable indicators of stress-responsive metabolic pathways (Albert et al., 2014; Wu et al., 2024). Among the genes implicated in PSRs, *SlMYB28* has been identified as a previously uncharacterized regulator of phosphate metabolism in tomato (Lin et al., 2025). As a key regulator of anthocyanin accumulation under phosphate deficiency, *SlMYB28* provides a suitable visual marker for validating the utility of our hairy root system in studying stress-responsive metabolic pathways.

In this study, we established a rapid and efficient RUBY-based hairy root transformation system for Micro-Tom, by systematically optimizing key parameters including *A. rhizogenes* strain, bacterial density (OD_600_), inoculation duration, AS concentration, and antibiotics for *Agrobacterium* eradication. To demonstrate the utility of this system for functional genomics, we applied it to characterize *SlMYB28* in tomato. Under the optimized conditions, the *35S:RUBY* control yielded a hairy root induction rate of 39.44% and a transformation efficiency of 66.01%, whereas *35S:SlMYB28* yielded 55.33% and 59.33%, respectively. This system provides a powerful platform for functional gene studies in tomato and lays the groundwork for similar systems in related Solanaceous species.

## 2 Materials and methods

### 2.1 Seed sterilization and germination

*Solanum lycopersicum* cv. Micro-Tom seeds were surface-sterilized with 1% sodium hypochlorite for 15 min, and rinsed three times with sterile distilled water. Sterilized seeds were cultured with agitation for two days. Germinated seedlings were transferred to Murashige and Skoog (MS) basal agar medium and grown at 25°C under standard white fluorescent light with 16-h light/8-h dark conditions. Ten days after sowing, tomato seedlings with fully expanded cotyledons were obtained for subsequent experiments.

### 2.2 Vector construction and *Agrobacterium*-mediated transformation

A vector containing the visually detectable RUBY reporter gene driven by the constitutive 35S promoter was used in this study. Six *A. rhizogenes* strains (ArQual, ArA4, C58C1, K599, MSU440 and Ar1193) were used for hairy root induction. *A. rhizogenes* competent cells were prepared according to a freeze-thaw protocol and transformed with the RUBY vector (Chen et al., 1994). Transformed colonies were selected on Tryptone-Yeast extract (TY) plates supplemented with the appropriate antibiotic (**Supplementary Table S1**). Glycerol stocks were prepared by suspension of fresh single colonies in liquid TY medium containing 50% (vol/vol) glycerol and stored at -80°C.

To evaluate the applicability of the optimized transformation system for functional gene studies, the full-length coding sequence (CDS) of *SlMYB28* was amplified from cDNA of tomato cv. Micro-Tom and cloned into a binary vector driven by the CaMV 35S promoter, generating the 35S::*SlMYB28* construct. The empty vector, lacking the *SlMYB28* insert, served as the negative control. Both constructs were introduced into the *A. rhizogenes* strain exhibiting the highest transformation efficiency via the aforementioned protocol, and were subsequently employed to induce transgenic hairy roots.

To further validate the function of *SlMYB28* in promoting anthocyanin accumulation, the same 35S::*SlMYB28* construct was introduced into *A. tumefaciens* strain GV3101 and used to generate stable transgenic tomato lines following a previously described protocol (Sun et al., 2006). Three independent homozygous transgenic lines (*SlMYB28-*OE-10, *SlMYB28-*OE-13, and *SlMYB28-*OE-14) were derived from separate transformation events, each originating from an independent cotyledon explant to ensure genetic independence. To determine homozygosity, T0 transgenic plants were self-pollinated to generate T1 progeny. Genomic DNA was extracted from individual T1 plants and subjected to PCR analysis using primers specific to the *SlMYB28* transgene and the selectable marker. The three lines used in this study (*SlMYB28-*OE-10, *SlMYB28-*OE-13, and *SlMYB28-*OE-14) were confirmed as homozygous by the absence of transgene segregation in at least 20 T2 progeny individuals per line, as verified by PCR.

### 2.3 In vitro hairy root transformation

Glycerol stocks of *A. rhizogenes* strains harboring the RUBY vector were suspended in 50 ml of TY liquid medium supplemented with the appropriate antibiotic (**Supplementary Table S1**) and incubated at 28°C, 200 rpm for 12 hours. Cells were pelleted by centrifugation at 5, 000 rpm for 5 minutes, re-suspended in MS liquid media (**Supplementary Table S2**), and adjusted to the desired optical density (OD_600_). Excised cotyledons (approximately 0.5 cm in length) were pre-cultured on MS basal medium (**Supplementary Table S2**) in darkness for 1 day before immersion in the bacterial suspension. Infected explants were blotted dry with sterile filter paper, and co-cultivated with the adaxial side down on co-cultivation medium (**Supplementary Table S2**) in darkness for 1–2 days.

### 2.4 Screening of optimal antibiotics and minimum effective concentrations for *A. rhizogenes* strains

After co-cultivation, explants were transferred to hairy root induction MS medium (**Supplementary Table S2**) supplemented with bacteriostatic antibiotics. Explants infected with *A. rhizogenes* strains ArQual, ArA4, or C58C1 were cultured on medium containing carbenicillin at 100, 200, 300, 400, 500, 600, or 700 mg/L to test the optimal concentration. Meanwhile, to screen for the optimal timentin concentration, explants infected with strains Ar1193, MSU440, and K599 were cultured on MS medium (**Supplementary Table S3**) containing timentin at 0, 100, 200, or 300 mg/L. Following a 10-day incubation period, bacteriostatic efficacy was assessed to determine the optimal antibiotic concentration for each of the six *A. rhizogenes* strains. Explant viability was assessed visually by observing the color and physical condition of the explants throughout the culture period. Explants were considered viable if they maintained a green appearance and showed no browning or necrosis at the cut edges. Explants exhibiting extensive browning, wilting, or loss of turgor were scored as non-viable.

### 2.5 Optimization of an *A. rhizogenes*-mediated transformation system

Based on a comparative analysis of the six *A. rhizogenes* strains, the strain exhibiting the highest transformation efficiency was identified as the most effective for infection. Furthermore, adjustment of key parameters, including infection duration, optical density of the bacterial suspension (OD_600_) and concentration of AS in the MS resuspension medium, were applied. Specifically, to determine the optimal infection duration, explants were inoculated with the best-performing *A. rhizogenes* strain suspension for 5, 10, or 15 min; to determine the optimal infection concentration, explants were infected with the best-performing *A. rhizogenes* strain resuspended at OD_600_ values of 0.5, 0.7, and 0.9; And to optimize AS concentration, the MS resuspension medium was supplemented with 0, 100, or 200 µmol/L AS prior to explant infection. The experiment followed a completely randomized design with three independent biological replicates, each consisting of one Petri dish containing 20–30 cotyledon explants. The Petri dish was treated as the experimental unit, and individual explants within the same dish were not considered statistically independent. The hairy root induction rate and the frequency of positive (RUBY-expressing) hairy roots were then calculated for each replicate.

### 2.6 Statistical analysis

Hairy root induction rate and transformation efficiency were determined by visual inspection of RUBY expression on the thirtieth day post-infection. For scoring of transgenic events, a hairy root was considered RUBY-positive if it displayed visible and uniform red pigmentation under white light. Roots with a length of less than 1 cm, or those showing only faint, speckled, or localized reddish spots, were excluded from the positive count to minimize the risk of false-positive scoring resulting from residual bacterial aggregates or transient RUBY expression. Representative RUBY-positive roots from each strain and construct were randomly selected and subjected to PCR analysis to confirm the presence of the RUBY transgene and the rolA/B/C markers, thereby verifying stable T-DNA integration. The HR and TE were then calculated using Equations 1 and 2 (Bian et al., 2022):

(1) $$\text{HR}\left(\%\right){\text{=N}}_{\text{hr}}{\text{/N}}_{\text{T}}\times 100$$

(2) $$\text{TE}\left(\%\right){\text{=N}}_{\text{RUBY}}{\text{/N}}_{\text{I}}\times 100$$

Where HR (hairy root induction rate) represents the proportion of explants that successfully produced hairy roots, reflecting the rhizogenic capacity of each *A. rhizogenes* strain. N_hr_ is the number of cotyledon explants bearing at least one hairy root (length ≥ 1 cm), and N_T_ is the total number of inoculated explants.

Where TE (transformation efficiency) represents the proportion of induced hairy roots that stably express the RUBY transgene, reflecting the T-DNA transfer and integration efficiency of each strain. N_RUBY_ is the number of RUBY-positive (red) hairy roots (length ≥ 1 cm), and N_I_ is the total number of hairy roots induced from all inoculated cotyledons (length ≥ 1 cm).

For the 35S::SlMYB28 overexpression construct, the same formulas (Equations 3, 4) were applied to calculate the hairy root induction rate and transformation efficiency:

(3) $$\text{HR}\left(\%\right){\text{=N}}_{\text{hr}}{\text{/N}}_{\text{T}}\times 100$$

(4) $$\text{TE}\left(\%\right){\text{=N}}_{\mathrm{MYB28}}{\text{/N}}_{\text{I}}\times 100$$

Where HR is the percentage of hairy root induction, N_hr_ is the number of cotyledons/seedlings with hairy roots (length ≥ 1 cm), N_T_ is the total number of inoculated cotyledons/infected seedlings, TE is transformation efficiency, N_MYB28_ is the number of hairy roots overexpressing the *MYB28* gene (length ≥ 1 cm), and N_I_ is the total number of hairy roots generated from inoculated cotyledons (length ≥ 1 cm).

It is important to distinguish the red pigmentation produced by the RUBY reporter from the purple pigmentation resulting from *SlMYB28* overexpression. RUBY drives betalain biosynthesis, yielding a red color in transgenic roots (He et al., 2020), whereas *SlMYB28* overexpression induces anthocyanin accumulation, which appears purple at vacuolar pH (Basílio et al., 2021; Stavenga et al., 2021).

The experiment followed a completely randomized design with three independent biological replicates, each consisting of one Petri dish containing 20–30 cotyledon explants. For each replicate (Petri dish), the HR and TE were calculated using the formulas described above, based on the explants and hairy roots within that dish. The percentage values obtained from the three independent replicates were then used to calculate the mean and standard deviation (SD) for each treatment. All statistical analyses were performed on these replicate-level percentage values (n = 3 per treatment). All data are presented as mean ± SD. Different lowercase letters indicate statistically significant differences at P<0.05.

### 2.7 Gene expression analysis by qRT-PCR

Total RNA was extracted using the FastPure Universal Plant Total RNA Isolation Kit (Vazyme Biotech Co., Ltd., Nanjing, China) according to the manufacturer’s instructions. First-strand cDNA was synthesized from 1 µg of total RNA using the HiScript IV RT SuperMix for qPCR (+gDNA wiper) (Vazyme Biotech Co., Ltd., Nanjing, China). Quantitative real-time PCR (qRT-PCR) was performed using gene-specific primers for SlMYB28 (**Supplementary Table S4**) on a real-time PCR detection system with SYBR Green master mix. The tomato Actin gene was used as the internal control for normalization. Relative expression levels were calculated using the 2^−ΔΔCT method. Three biological replicates were analyzed for each sample.

For expression analysis in stable transgenic lines, total RNA was extracted from leaves of wild-type (WT) and three independent 35S:SlMYB28 overexpression lines (SlMYB28-OE-10, SlMYB28-OE-13, and SlMYB28-OE-14) at 30 days after germination (DAG). For expression analysis in transgenic hairy roots, total RNA was extracted from SlMYB28-overexpressing and empty vector-transformed hairy roots at 30 days post-infection. Statistical significance for the stable transgenic lines was determined using one-way ANOVA with Tukey’s test (different lowercase letters indicate significant differences at P< 0.05), while comparisons between hairy root samples were analyzed using Student’s t-test.

### 2.8 Anthocyanin content measurement

To evaluate anthocyanin accumulation in transgenic lines, we measured the anthocyanin content in the aerial parts of wild-type (WT) and three independent 35S:SlMYB28 overexpression lines (SlMYB28-OE-10, SlMYB28-OE-13, and SlMYB28-OE-14) at six developmental time points (10, 20, 30, 40, 50, and 60 days after germination), following the protocol described by Zhang et al. (2019) with minor modifications. Approximately 100 mg fresh weight of aerial tissues (including stems and leaves) was harvested from each line at each time point and placed into 7 mL centrifuge tubes. The samples were extracted with 2.1 mL of methanol containing 1% (v/v) HCl at 4 °C in the dark for 24 h. Subsequently, 1.4 mL of chloroform and 1.4 mL of distilled water were sequentially added to each tube, and the mixtures were thoroughly vortexed to remove chlorophyll. After centrifugation for 10 min at 4 °C, the upper aqueous phase was carefully collected, and its absorbance at 530 nm (OD_530_) was measured using a UV-visible spectrophotometer. Anthocyanin content was calculated as OD_530_ per gram fresh weight (OD_530_/g FW). Three biological replicates were analyzed for each line at each time point.

## 3 Results

### 3.1 Determination of minimal effective antibiotic concentrations for eliminating residual *A. rhizogenes* strains

Following *A. rhizogenes* inoculation, cotyledon explants were cultured on hairy root induction medium supplemented with antibiotics to eradicate residual bacteria. Insufficient antibiotic concentrations fail to eliminate residual *Agrobacterium* completely, resulting in explant browning, necrosis, and reduced transformation efficiency. Therefore, determination of the optimal antibiotic concentration for each *A. rhizogenes* strain is essential. Through systematic gradient screening (**Supplementary Table S3**), the minimum effective concentrations (MECs) of antibiotics required to eradicate each of the six strains without compromising explant viability were determined in the hairy root induction MS medium (**Supplementary Table S2**; **Table 1**). The MECs were as follows: 300 mg/L carbenicillin for ArQual, 200 mg/L carbenicillin for ArA4, 600 mg/L carbenicillin for C58C1, and 100 mg/L Timentin for K599, MSU440, and Ar1193.

**Table 1:** Minimum effective concentrations (MECs) of antibiotics for different *A. rhizogenes* strains.

*A. rhizogenes* strain	MECs of antibiotics
Ar Qual	300mg/L carbenicillin
Ar A4	200mg/L carbenicillin
C58C1	600mg/L carbenicillin
K599	100mg/L timentin
MSU440	100mg/L timentin
Ar 1193	100mg/L timentin

Screening of antibiotic concentrations was performed under standardized conditions: *A. rhizogenes* suspension at an OD_600_ of 0.7, inoculation duration 10 min, MS resuspension medium with 100 μM acetosyringone (AS), and dark co-cultivation 2 days. The above conditions represent the standardized conditions used during the antibiotic screening stage and do not constitute the final optimized parameters. Three biological replicates (Petri dishes), 20–30 cotyledon explants per replicate.

### 3.2 Evaluation of different *A. rhizogenes* strains for hairy root induction in tomato cotyledon explants

The efficiency of hairy root induction is well documented to be strain-dependent in *A. rhizogenes*-mediated transformation. Therefore, to identify the most suitable strain for MicroTOM, we evaluated six strains (ArQual, ArA4, C58C1, K599, MSU440, and Ar1193) for their ability to induce hairy roots from cotyledon explants. At 30 days post-inoculation, five of the six strains (ArQual, ArA4, K599, MSU440, and Ar1193) successfully induced *RUBY*-expressing (red) hairy roots from the inoculation sites, whereas the C58C1 strain completely failed to induce hairy roots (0% transformation efficiency), exhibiting only callus formation and shoot regeneration (**Figure 1A**; **Table 2**). Notably, both RUBY-positive (red) and RUBY-negative (white) hairy roots frequently emerged from the same explant, although some explants produced exclusively red or white hairy roots.

**Figure 1:**
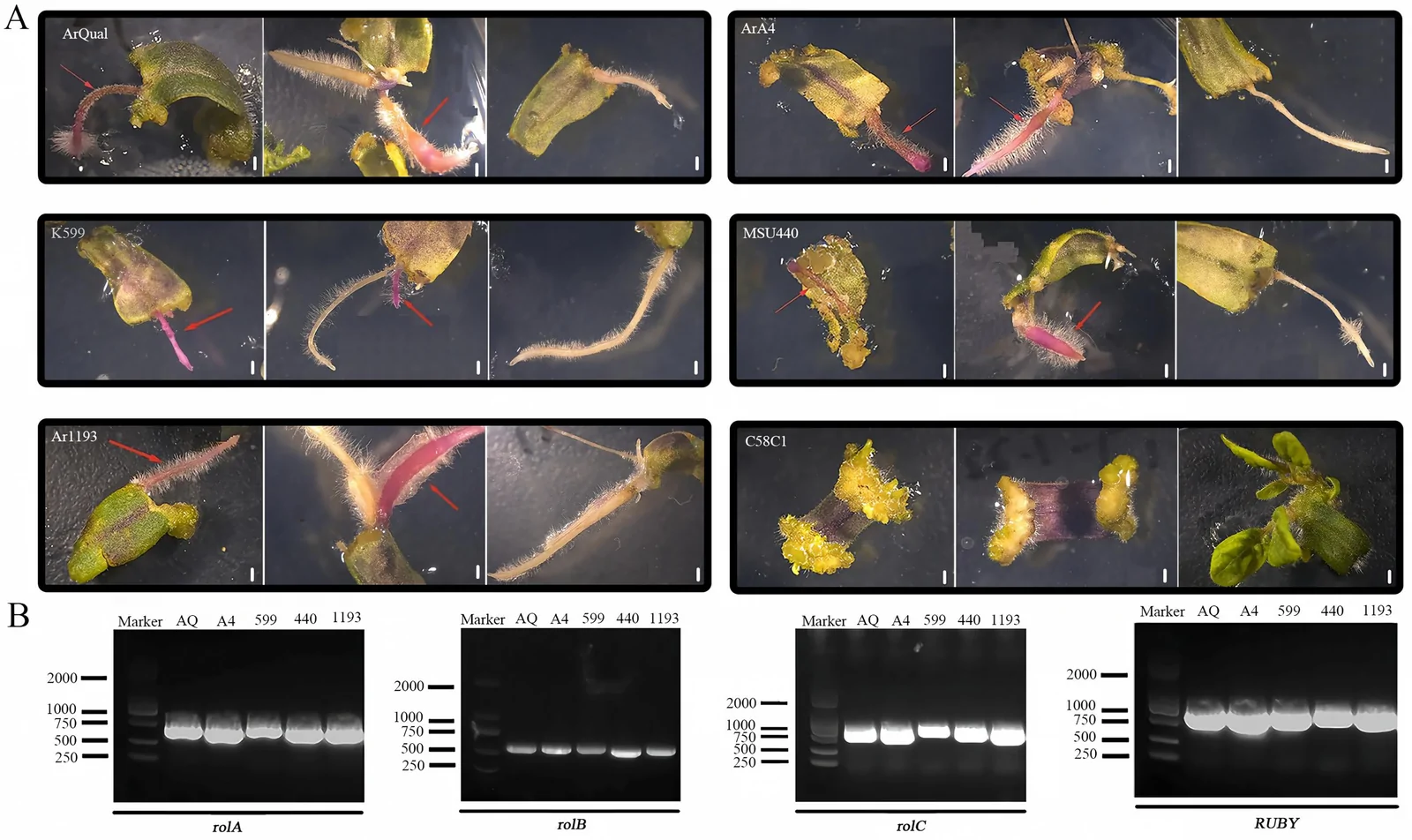
Hairy root induction from tomato cotyledons and molecular identification of RUBY-expressing hairy roots. **(A)** Hairy roots induced from individual cotyledons at 30 days post-inoculation with six *A. rhizogenes* strains. Scale bar = 1 mm. **(B)** PCR identification of RUBY-expressing hairy roots induced by five *A. rhizogenes* strains (ArQual, ArA4, K599, MSU440, and Ar1193). M, DNA marker (DL 2000 bp); rolA (525 bp); rolB (423 bp); rolC (626 bp); RUBY (763 bp).

**Table 2:** Hairy root induction rates and transformation efficiencies of six *A. rhizogenes* strains in Micro-Tom cotyledon explants.

*A. rhizogenes* strain	Number of explants	Number of explants producing hairy roots	Total number of hairy roots induced	Number of positive (RUBY-expressing) hairy roots	Hairy root induction rate (%)	Mean hairy root induction rate (%)	Positive hairy root induction rate (%)	Mean positive hairy root induction rate (%)
Ar Qual	22	6	7	6	27.27	26.03 ± 4.71^b^	85.71	61.90 ± 20.62^a^
	20	6	10	5	30.00		50.00	
	24	5	6	3	20.83		50.00	
Ar A4	30	16	51	1	53.33	63.61 ± 9.14^a^	0.20	3.41 ± 3.04^b^
	21	14	32	2	66.67		6.25	
	24	17	53	2	70.83		3.77	
C58C1	22	0	0	0	0.00	0.00 ± 0.00^d^	0.00	0.00 ± 0.00^b^
	20	0	0	0	0.00		0.00	
	23	0	0	0	0.00		0.00	
K599	30	1	3	1	3.33	4.01 ± 4.39^cd^	33.33	27.78 ± 25.46^ab^
	23	2	2	1	8.70		50.00	
	28	0	0	0	0.00		0.00	
MSU440	31	4	7	1	12.90	16.53 ± 12.49^bc^	14.29	41.13 ± 51.05^ab^
	23	7	11	1	30.43		9.09	
	32	2	1	1	6.25		100.00	
Ar 1193	30	6	12	4	20.00	16.51 ± 6.04^bc^	33.33	24.89 ± 14.62^ab^
	21	2	3	1	9.53		33.33	
	30	6	26	2	20.00		8.00	

The values are presented as the mean ± SD of three independent biological replicates (n = 3, with each replicate consisting of one Petri dish containing 20–30 explants). Different alphabetical letters indicate statistical significance at p<0.05 (one-way ANOVA with Tukey’s test).

To confirm that the red coloration resulted from *RUBY* transgene expression, genomic DNA was extracted from red hairy roots induced by the five *A. rhizogenes* strains and analyzed by PCR. The hairy root-specific markers *rolA*, *rolB*, and *rolC*, as well as the RUBY transgene, were detected in all red hairy roots examined (**Figure 1B**). These results confirmed that hairy roots could be successfully induced in Micro-Tom, and that the *RUBY* reporter gene was expressed in the transformed roots, thereby facilitating further optimization of the transformation system.

Hairy root induction rates and transformation efficiencies for the six strains are summarized in **Table 2**. The induction rates ranked as follows: ArA4 > ArQual > MSU440 > Ar1193 > K599 > C58C1, averaging 63.61%, 26.03%, 16.53%, 16.51%, 4.01%, and 0.00%, respectively, while the transformation efficiencies ranked as follows: ArQual > MSU440 > K599 > Ar1193 > ArA4 > C58C1, at 61.90%, 41.13%, 27.78%, 24.89%, 3.41%, and 0.00%, respectively. Among the six strains, ArA4 exhibited the highest hairy root induction rate (63.61%), whereas ArQual showed the highest transformation efficiency (61.90%). Although ArQual had a lower overall hairy root induction rate than ArA4, it yielded the highest frequency of *RUBY*-positive hairy roots. Therefore, ArQual was selected as the optimal *A. rhizogenes* strain for subsequent inoculation experiments.

### 3.3 Optimization of transformation parameters for *A. rhizogenes* strain ArQual

To optimize the transformation efficiency of strain ArQual, we evaluated the following key parameters: bacterial suspension density (OD_600_), inoculation duration, and AS concentration. Cotyledon explants from 10-day-old seedlings were inoculated, and transformation efficiency was calculated as the proportion of RUBY-positive hairy roots.

We first examined the effects of infection duration (5, 10, and 15 min) on hairy root induction and transformation efficiency. Both parameters increased from 5 to 10 min and reached their maxima at 10 min, but declined sharply thereafter (**Figure 2A**). Strikingly, neither hairy root induction nor transformation was detected at 15 min, with both values dropping to zero (**Figure 2B**). These results identify 10 min as the optimal infection duration for ArQual. Under the optimized 10-min infection condition, we subsequently assessed the effects of bacterial cell density on hairy root induction and transformation efficiency. A gradient of *Agrobacterium* suspensions ranging from an OD_600_ of 0.5 to 0.9 was tested (**Figure 2C**). Both the hairy root induction rate and transformation efficiency were significantly higher at an OD_600_ of 0.7 than at the other two concentrations, reaching 30.91% and 59.44%, respectively (**Figure 2D**). These results establish an OD_600_ of 0.7 as the optimal cell density for ArQual-mediated infection of tomato cotyledons. Furthermore, under the optimized conditions of a 10 min infection duration and an OD_600_ of 0.7, we evaluated the effect of AS concentration on transformation efficiency. Increasing AS from 0 to 100 µM resulted in a slight, albeit non-significant, elevation in hairy root induction rate; however, the transformation efficiency at 100 µM was significantly higher than that at 0 µM. Upon further increasing AS to 200 µM, both hairy root induction and transformation efficiency were significantly elevated compared to 0 µM, reaching mean values of 39.44% and 66.01%, respectively (**Figures 2E, F**). These results indicate that AS supplementation enhances ArQual-mediated transformation, with 200 µM representing the optimal concentration under the tested conditions.

**Figure 2:**
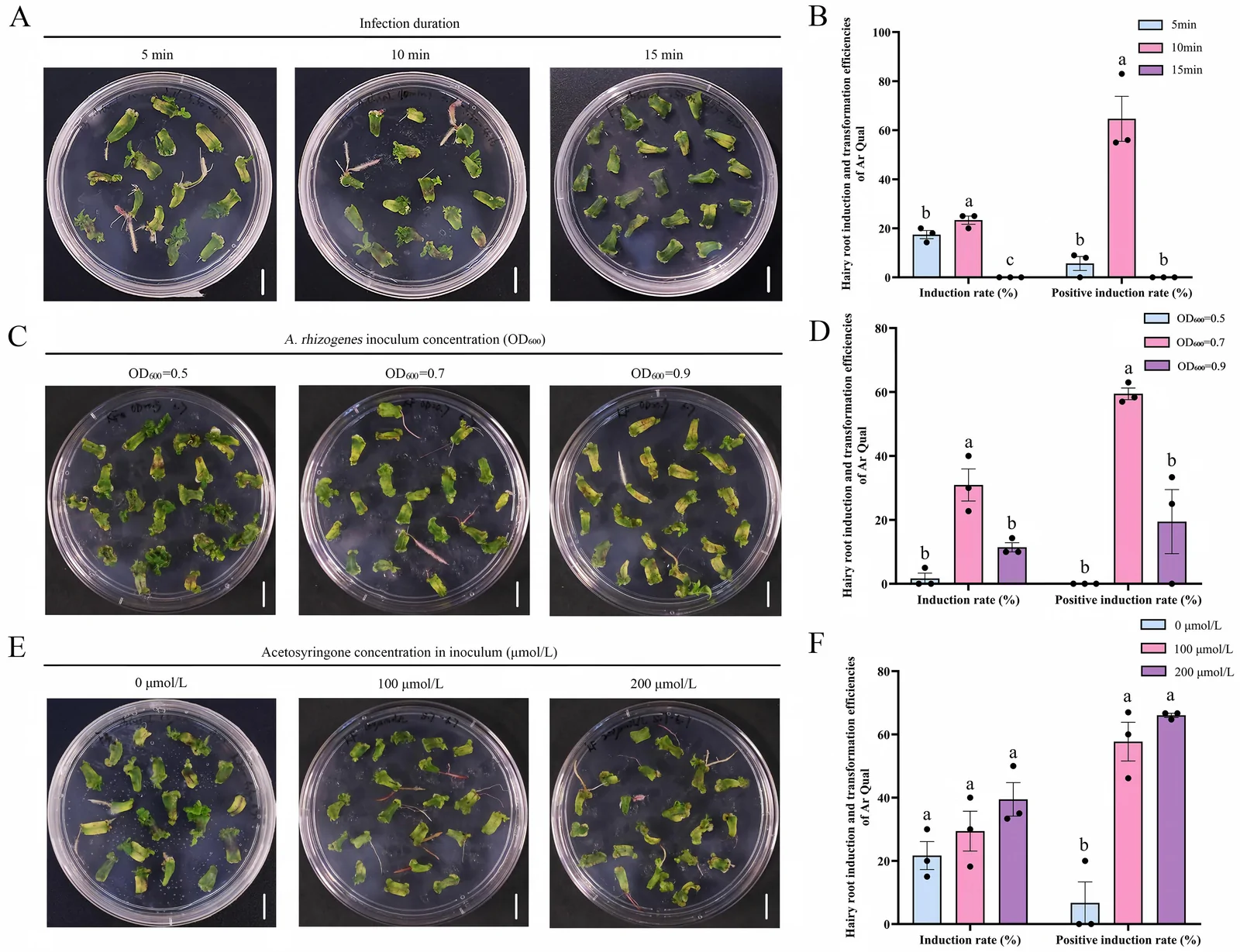
Optimization of transformation parameters for *A. rhizogenes* strain ArQual in Micro-Tom. **(A)** Representative images of tomato cotyledon explants following infection with ArQual for 5, 10, and 15 min. Scale bar = 1 cm. **(B)** Hairy root induction rates and transformation efficiencies under the indicated infection durations. **(C)** Representative images of tomato cotyledon explants infected with ArQual at OD_600_ = 0.5, 0.7, and 0.9. Scale bar = 1 cm. **(D)** Hairy root induction rates and transformation efficiencies at the indicated bacterial cell densities. **(E)** Representative images of tomato cotyledon explants infected with ArQual in the presence of 0, 100, and 200 µM acetosyringone (AS). Scale bar = 1 cm. **(F)** Hairy root induction rates and transformation efficiencies at the indicated AS concentrations. Values represent means ± SD of three biological replicates. Different lowercase letters denote significant differences at p< 0.05.

Collectively, the optimized transformation parameters were established as follows: *Agrobacterium rhizogenes* cell suspension at an OD_600_ of 0.7, an infection duration of 10 min, and an AS concentration of 200 µM.

### 3.4 Validation of *SlMYB28* function using the optimized hairy root transformation platform

To further assess the utility of our optimized hairy root transformation system for functional gene studies, we applied it to validate the role of *SlMYB28* in anthocyanin accumulation. The involvement of *SlMYB28* in this process was previously demonstrated by reduced anthocyanin levels in the *slmyb28* mutant under phosphate deficiency stress (Lin et al., 2025). To examine the effect of *SlMYB28* overexpression, we generated three *35S:SlMYB28* stable transformation lines (*SlMYB28-*OE-10, *SlMYB28-*OE-13, and *SlMYB28-*OE-14). RT-qPCR analysis revealed significantly higher *SlMYB28* transcript levels in all three overexpression lines relative to WT, with *SlMYB28-*OE-10 showed the highest expression level, followed by *SlMYB28-*OE-13, and *SlMYB28-*OE-14 exhibited the lowest transcript level (**Figure 3A**). When germinated on 1/2 MS medium, the *SlMYB28-*OE-10 line, which exhibited the highest *SlMYB28* transcript level (**Figure 3A**), displayed obvious purple pigmentation in both stems and leaves at 5 days after germination, with the purple coloration in root tips being particularly prominent against the white background. In contrast, no visible purple pigmentation was observed in the WT (**Figure 3B**). To examine the phenotypic effects further, we measured the anthocyanin content in plants at 10 days after germination (**Figures 3C, D**). The result showed that all three overexpression lines exhibited higher anthocyanin levels than WT. Notably, the *SlMYB28*-OE-10 line, which had the highest *SlMYB28* expression, displayed significantly increased anthocyanin accumulation with visible purple pigmentation in the plant (**Figures 3C, D**). These results further support the positive regulatory role of *SlMYB28* in anthocyanin biosynthesis.

**Figure 3:**
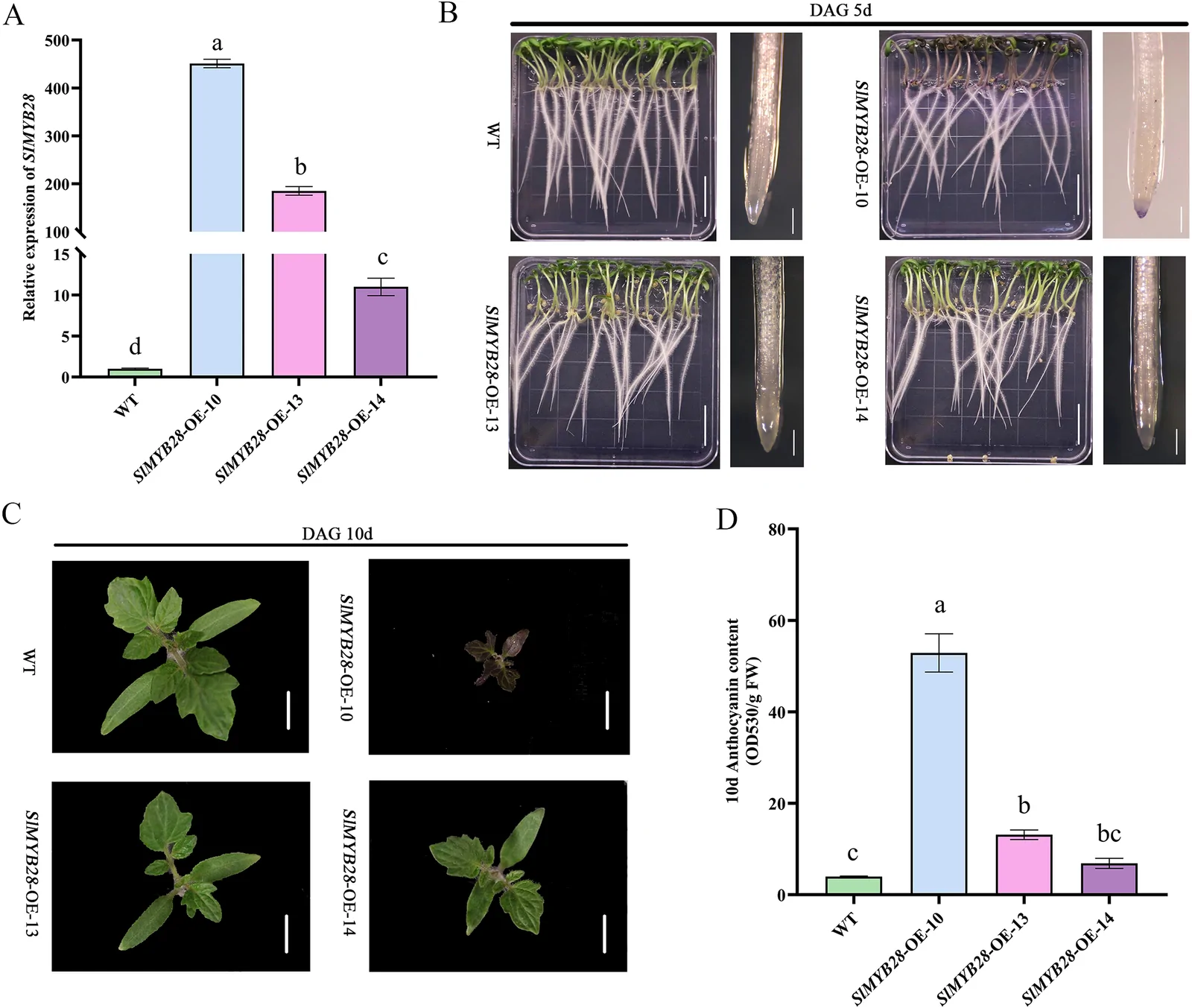
Overexpression of *SlMYB28* promotes anthocyanin accumulation in tomato. **(A)** Relative expression of *SlMYB28* in wild-type (WT) and three 35S:*SlMYB28* overexpression lines (*SlMYB28*-OE-10, *SlMYB28*-OE-13, and *SlMYB28*-OE-14) determined by qRT-PCR. Values represent means ± SD of three biological replicates. Different lowercase letters indicate significant differences at P< 0.05 (one-way ANOVA with Tukey’s test). **(B)** Phenotypes of WT and *SlMYB28*-overexpressing lines at 5 days after germination on 1/2 MS medium. Scale bars: 2 cm (left) and 0.5 mm (right). **(C)** Representative growth phenotypes of WT and SlMYB28-overexpressing lines at 10 days after germination. Scale bars = 1 cm. **(D)** Anthocyanin content in aerial parts of WT and *SlMYB28*-overexpressing lines at 10 days after germination. Values represent means ± SD of three biological replicates. FW, fresh weight.

Next, to demonstrate that our optimized hairy root system enables rapid and reliable functional validation, we applied it to independently assess the role of *SlMYB28* in anthocyanin accumulation. Specifically, we cloned the *SlMYB28* coding sequence into a 35S-driven binary vector, and the resulting construct-together with the empty vector control-was introduced into *A. rhizogenes* strain ArQual to generate transgenic hairy roots following the optimized protocol. Representative images of empty vector-transformed hairy roots and *SlMYB28*-overexpressing hairy roots at 30 days post-infection are shown in **Figure 4A, B**, respectively. The mean induction rate and transformation efficiency for *SlMYB28* overexpression were 55.33% and 59.33%, respectively (**Figure 4D**). PCR analysis confirmed the presence of the *SlMYB28* transgene and the hairy root-specific markers *rolA*, *rolB*, and *rolC* in overexpression lines, whereas only the rol genes were detected in the empty vector controls (**Figure 4C**), confirming successful integration of the *35S:SlMYB28* construct. RT-qPCR analysis further revealed significantly elevated *SlMYB28* expression in overexpression roots compared with controls (**Figure 4E**). Notably, empty vector-transformed hairy roots remained white, whereas *SlMYB28*-overexpressing hairy roots exhibited a purple phenotype (**Figures 4A, B**). These results are consistent with the reduced anthocyanin level in the mutant of *SlMYB28*, and further support the role of *SlMYB28* in promoting anthocyanin accumulation in tomato. Collectively, these findings establish the *A. rhizogenes*-mediated hairy root system as an effective platform for rapid functional analysis in tomato (**Figure 5**).

**Figure 4:**
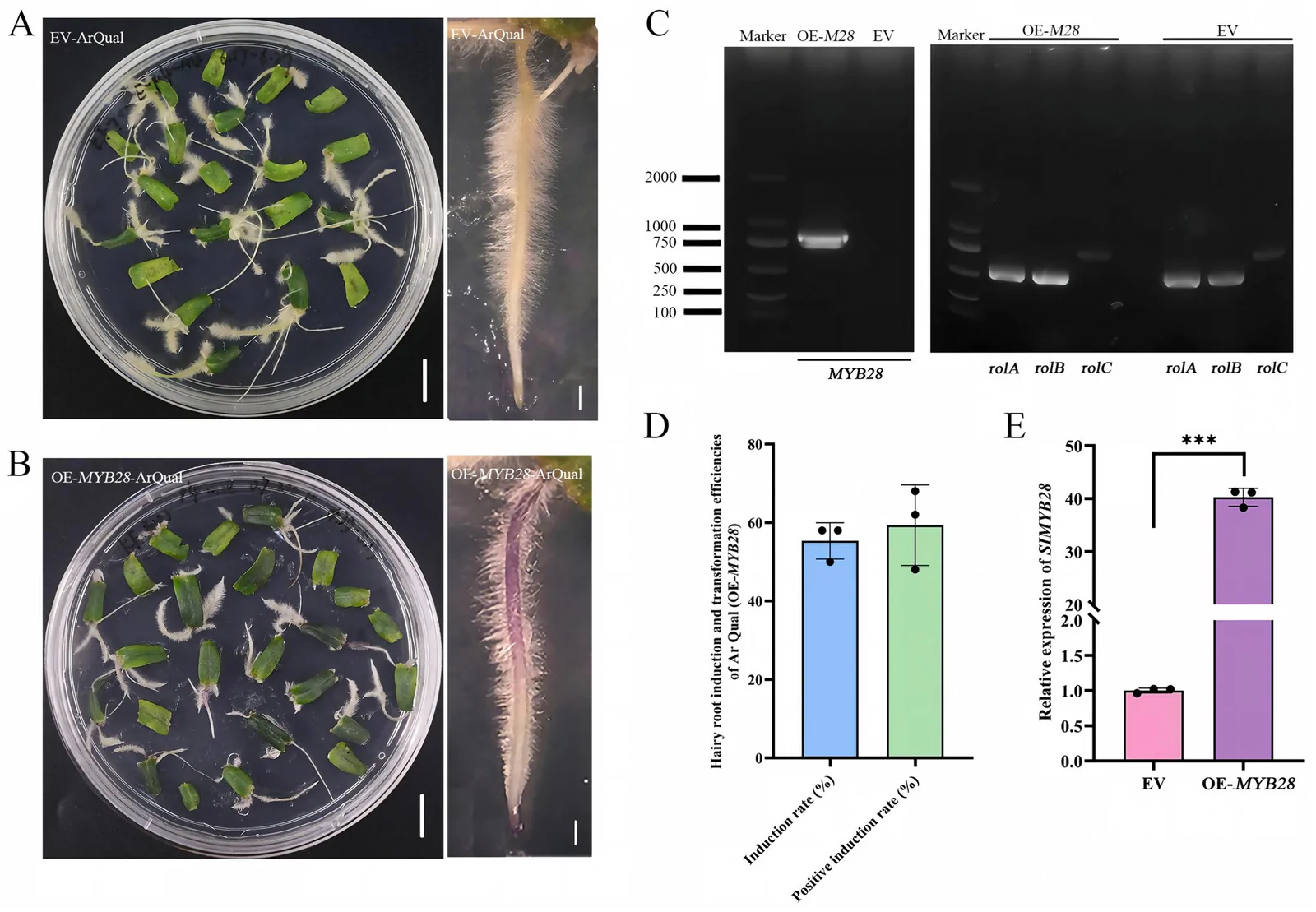
Functional characterization of *SlMYB28* via *A. rhizogenes*-mediated hairy root transformation in tomato. **(A)** Hairy roots induced by the empty vector control (EV-ArQual) in tomato cotyledon explants. Scale bars:=1 cm **(left)** and 1 mm (right). **(B)** Hairy roots induced by the *SlMYB28* overexpression construct (OE-*MYB28*-ArQual) in tomato cotyledon explants, showing purple pigmentation. Scale bars: 1 cm (left) and 1 mm (right). **(C)** PCR identification of transgenic hairy roots. M: DL 2000 bp DNA marker; lanes labeled OE-M28 and EV indicate hairy roots induced by OE-*SlMYB28*-ArQual and EV-ArQual, respectively. The *SlMYB28* transgene (735 bp) and the hairy root-specific markers rolA (525 bp), rolB (423 bp), and rolC (626 bp) were amplified. **(D)** Hairy root induction rate and transformation efficiency of tomato cotyledon explants transformed with OE-*SlMYB28*-ArQual. **(E)** Relative expression of *SlMYB28* in hairy roots induced by EV-ArQual and OE-*SlMYB28*-ArQual, as determined by RT-qPCR. Data represent the mean ± SD of three biological replicates. Asterisks indicate significant differences at P< 0.05 (Student’s *t*-test).

**Figure 5:**
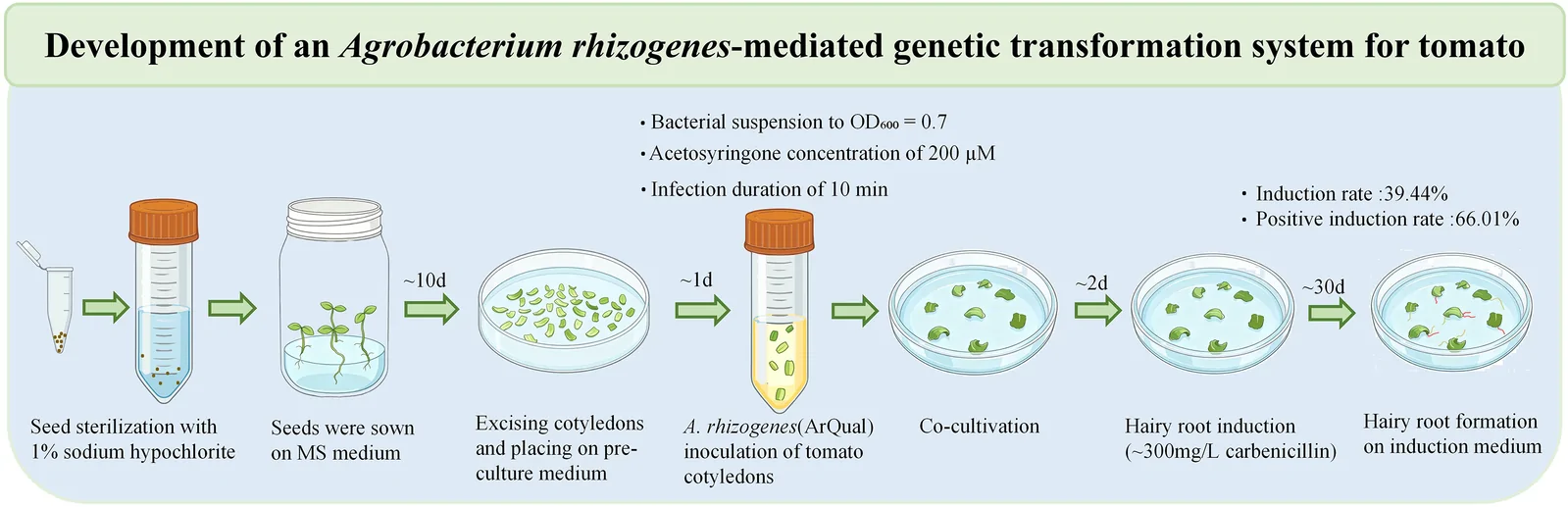
Schematic overview of the *A. rhizogenes*-mediated transformation protocol for tomato cv. Micro-Tom. The diagram depicts the process from explant preparation to hairy root induction using *A. rhizogenes* strain ArQual. The optimized parameters are annotated within the workflow: bacterial resuspension density at an OD_600_ of 0.7, acetosyringone (AS) concentration of 200 µM, infection duration of 10 min, and carbenicillin concentration of 300 mg/L for hairy root induction.

## 4 Discussion

*Agrobacterium tumefaciens*-mediated stable transformation remains the standard approach for generating transgenic tomato plants for whole-plant functional studies. However, this method is time-consuming and labor-intensive, typically requiring 4–6 months from explant inoculation to the acquisition of T0 transgenic plants, with an additional 1–2 generations of self-pollination needed to obtain homozygous lines (Kassahun et al., 2021; Pagliuso et al., 2025). Transformation efficiencies vary considerably depending on genotype, explant type, bacterial strain, and culture conditions (Nonaka et al., 2019; Arias-G and Uribe, 2025; Pagliuso et al., 2025). In contrast, *Agrobacterium rhizogenes*-mediated hairy root transformation enables the rapid production of transgenic roots suitable for phenotypic analysis within a short timeframe (Ajdanian and Torkamaneh, 2025; Liu et al., 2026), and allows for the identification of individual transformed clones using selectable marker genes. These characteristics have made this technique widely applicable to gene function analysis (Gutierrez-Valdes et al., 2020), investigation of plant transformation events (Aggarwal et al., 2018), and secondary metabolism research (Cui et al., 2020). While hairy root transformation is restricted to root tissues and does not replace stable transformation for whole-plant functional analysis, it offers a rapid, efficient, and cost-effective alternative for initial functional screening of root-expressed genes. In tomato, several *A. rhizogenes* strains, including A4, R1000, ATCC 15834, MSU440, K599, ArQual, and C58C1, have been employed for hairy root transformation in various cultivars (Ron et al., 2014; Bian et al., 2022). For instance, MSU440, K599, ArQual, and C58C1 have been reported to induce hairy roots in cv. Ailsa Craig (AC) (Bian et al., 2022); K599 has been utilized in cv. M82 for function studies of the RolB protein (Gryffroy et al., 2023); and MSU440 has been applied for hairy root induction in cv. Moneymaker and wild tomato *S. pennellii* (Ron et al., 2014). However, despite these advances in other cultivars, the application of *A. rhizogenes*-mediated transformation in Micro-Tom remains notably underdeveloped.

To date, the application of *A. rhizogenes*-mediated transformation in Micro-Tom has been largely limited to individual strains without systematic parameter optimization (Ho-Plágaro et al., 2018; Tóth et al., 2022). For instance, Ho-Plágaro et al. (2018) employed MSU440 specifically for studying arbuscular mycorrhizal symbiosis, while Tóth et al. (2022) utilized ArQual for functional analysis of root-specific resistance genes-neither of which involved optimization of the *A. rhizogenes*-mediated hairy root transformation system. This gap represents a significant technical bottleneck that constrains the application of this powerful approach in functional studies of this model tomato cultivar. Our study distinguishes itself from these earlier efforts by providing a comprehensive, multi-parameter optimization that covers strain selection, antibiotic sensitivity profiling, infection duration, bacterial density, and AS concentration, all integrated with the RUBY-based visual screening system. This systematic approach not only enhances transformation efficiency but also establishes a reproducible and user-friendly platform that was previously unavailable for Micro-Tom.

In this study, effective suppression of bacterial overgrowth is a critical prerequisite for obtaining axenic hairy roots; however, different *Agrobacterium* strains exhibit markedly different sensitivities to the same antibiotic (Niazian et al., 2023; Xu et al., 2023). Carbenicillin and timentin are two major antibiotics used for suppressing the residual bacteria in previous studies (Yang et al., 2015; de Koning et al., 2023; Niazian et al., 2023; Ma et al., 2025). Through systematic gradient screening (**Supplementary Table S3**), we determined the minimum effective concentrations (MECs) of carbenicillin or timentin for the six strains (**Table 1**), which reveals substantial inter-strain variation. For example, ArA4 was inhibited by 200 mg/L carbenicillin, whereas C58C1 required 600 mg/L. Following the determination of MECs, we assessed the efficiency of different *A. rhizogenes* strains in mediating hairy root induction and gene transformation (**Table 2**). As shown in **Table 1** and **2**, the six *A. rhizogenes* strains exhibited differential performance, which may arise from multiple factors. First, these strains harbor distinct *Ri* plasmid types, which differ in their T-DNA structure and the presence of auxin biosynthesis genes (*iaaM*, *iaaH*) and opine synthesis genes (*ags*, *mas*, *ocs*). For instance, agropine-type strains (e.g., ArQual, ArA4, C58C1, MSU440) typically carry two T-DNAs (TL- and TR-DNA) and are considered highly virulent, whereas mannopine-type strains (e.g., K599) and cucumopine-type strains (e.g., Ar1193) possess different T-DNA configurations that may affect both rhizogenic capacity and transgene integration efficiency (Veena and Taylor, 2007; Gantait and Mukherjee, 2021). Second, variation in vir gene expression levels and regulatory functions among strains can influence the efficiency of T-DNA processing and nuclear import, ultimately affecting stable transformation rates (Oger et al., 2004; Gelvin, 2012). Furthermore, the distinct antibiotic sensitivity profiles we observed (**Table 1**) represent another contributing factor. Strains that require higher antibiotic concentrations—such as C58C1, which needed 600 mg/L carbenicillin—may exhibit reduced transformation efficiency not only due to poor virulence but also because of antibiotic-induced phytotoxicity that can compromise explant viability during the selection period (Nauerby et al., 1997; Cheng et al., 1998).

Among the tested strains, ArQual exhibited the highest transformation efficiency in Micro-Tom, consistent with previous findings in cv. Ailsa Craig (Bian et al., 2022). Notably, ArA4 showed the highest hairy root induction rate (63.61%) but an extremely low transformation efficiency (3.41%), whereas ArQual achieved the highest transformation efficiency (61.90%) despite a lower induction rate (**Table 2**). This dissociation between rhizogenic capacity and T-DNA transfer efficiency has been widely documented (Bian et al., 2022; Niazian et al., 2023), and similar cases have been reported in other species such as chrysanthemum (Tsuro et al., 2005). It is also possible that some hairy roots induced by ArA4 contained the T-DNA but did not express RUBY at a level sufficient for visible red pigmentation, and were therefore not counted as positive. The superior performance of ArQual likely reflects specific compatibility with the Micro-Tom cultivar, as strain–host specificity is well documented in *Agrobacterium*–plant interactions (Anderson and Moore, 1979).

Furthermore, we optimized three key factors in the hairy root transformation system, including infection duration, bacterial suspension density(OD_600_), and AS concentration to maximize the transformation efficiency in Micro-Tom. Infection duration determines the extent of bacterial contact with wounded explant tissues and directly affects T-DNA transfer frequency. However, prolonged infection may lead to bacterial overgrowth, causing phytotoxicity and subsequent explant necrosis (Fan et al., 2020). Our results established 10 min as the optimal infection duration for Micro-Tom (**Figure 2B**), and revealed a pattern consistent with observations in other plant species, such as *Solanum nigrum* (Zhang et al., 2026) and cucumber (Fan et al., 2020), where extended infection periods similarly reduced hairy root induction, underscoring the existence of a narrow optimal window for efficient *A grobacterium*-mediated transformation. Bacterial suspension density (OD_600_) determines the number of Agrobacterium cells attaching to wound sites and subsequent T-DNA delivery (Chen et al., 2018; Niazian et al., 2023). In our study, an OD_600_ of 0.7 yielded significantly higher induction and transformation rates compared to lower (0.5) or higher (0.9) densities (**Figure 2D**), mirroring density-dependent responses reported in other plant species such as blueberry, where optimal transformation occurred at and OD_600_ of 1.0 and decreased at higher densities (Wu et al., 2025). AS induces *Agrobacterium vir* gene expression and significantly enhances transformation efficiency (Biswas et al., 2023). We found that increasing AS concentration did not affect hairy root induction rate but markedly improved the hairy root transformation rate, a trend consistent with the optimized systems in blueberry, soybean, and *Echium khuzistanicum* (Niazian et al., 2023; Mohammadi et al., 2024; Wu et al., 2025). Collectively, these results demonstrate that fine-tuning infection duration, bacterial density, and AS concentration is essential for achieving high transformation efficiency while maintaining explant viability in Micro-Tom.

*SlMYB28* is a member of the MYB transcription factor family that integrates environmental stress signals with metabolic regulation (Wu et al., 2024). Previous studies have shown that the anthocyanin content is reduced in the *slmyb28* mutant, suggesting its potential role in regulating this important stress-responsive metabolite (Lin et al., 2025). To confirm this regulatory function prior to hairy root-based analysis, we generated transgenic tomato lines overexpressing *SlMYB28*. Our results showed that *SlMYB28* promotes anthocyanin accumulation, with the highest-expressing line (*SlMYB28-*OE-10) exhibiting significantly increased anthocyanin content and visible purple pigmentation (**Figure 3**). This finding is consistent with the threshold-dependent mode of action commonly observed for MYB activators of anthocyanin biosynthesis (Albert et al., 2014). Notably, the most conspicuous purple coloration was observed in the root tips of *SlMYB28-*OE-10seedlings at 5 days after germination (**Figure 3B**), suggesting that *SlMYB28* may serve as a suitable reporter gene to validate the utility of the hairy root transformation system.

To test this, we applied the optimized hairy root transformation system to validate the function of *SlMYB28* in tomato. Using the 35S:*SlMYB28* construct, we achieved a transformation efficiency of 59.33% (**Figure 4D**). Notably, transformation efficiency inherently varies among species, as evidenced by the reported efficiencies of approximately 51.7% in apple (Liu et al., 2024) and 47.4% in blueberry (Wu et al., 2025). Overexpression of *SlMYB28* resulted in a distinct purple pigmentation in transgenic roots, whereas empty vector controls remained white (**Figures 4A, B**). This phenotype reflects vacuolar anthocyanin accumulation, predominantly present as purple quinoidal bases at vacuolar pH 4-6 (Basílio et al., 2021; Stavenga et al., 2021), and provides high-contrast visual confirmation of *SlMYB28*’s role in promoting anthocyanin accumulation, consistent with its previously reported involvement in phosphate deficiency responses (Lin et al., 2025). This observation aligns with the result of Massa et al. (2022), who reported similar anthocyanin accumulation upon overexpression of *AtMYB75* in tomato hairy roots, and underscores the value of our system for rapid visual screening of genes involved in stress-responsive metabolic pathways.

While the hairy root system offers clear advantages for rapid functional screening, it is important to recognize its inherent limitations. First, this system is restricted to root tissues and does not enable functional analysis of genes in aerial organs such as leaves, flowers, or fruits. Second, hairy roots are induced by *A. rhizogenes* and carry the Ri T-DNA, which includes *rol* genes that can alter root development, hormone metabolism, and secondary metabolite profiles (Gelvin, 2003; Bahramnejad et al., 2019). Although we confirmed the presence of *rolA/B/C* markers in transgenic roots (**Figures 1B**, **4C**), the potential effects of these Ri-derived genes on root physiology and metabolism should be considered when interpreting phenotypes. Third, hairy roots differ from normal roots in their origin (wound-induced versus seed-borne) and developmental context, which may affect their metabolic and transcriptional responses. Additionally, PCR validation of transgene integration was performed on representative RUBY-positive roots rather than on every individual root, and thus a small proportion of roots with weak or variable RUBY expression may have been missed in the visual scoring. Consequently, phenotypes observed in hairy roots may not fully recapitulate those in intact plants, and validation in stable transgenic lines remains essential for confirming gene function at the whole-plant level. Despite these limitations, the hairy root system remains a valuable tool for initial functional screening due to its speed, efficiency, and visual tractability.

In conclusion, we have established an optimized *A. rhizogenes*-mediated hairy root transformation system for *Solanum lycopersicum* cv. Micro-Tom through systematic evaluation of antibiotic sensitivity, strain selection, infection duration, bacterial density, and AS concentration (**Figure 5**). The protocol achieves high transformation efficiency and provides an efficient platform for rapid functional screening and preliminary functional characterization in Micro-Tom. Its practical utility is exemplified by the rapid validation of *SlMYB28*’s role in anthocyanin accumulation, demonstrating the system’s potential application for investigating genes involved in stress-responsive metabolic pathways. This approach offers a simple, efficient, and visually tractable tool for functional genomics research in tomato.

## Data Availability

The original contributions presented in the study are included in the article/**Supplementary Material**. Further inquiries can be directed to the corresponding authors.
