# Supplementary material for: Establishment of an efficient Agrobacterium rhizogenes-mediated hairy root transformation system for functional analysis in tomato
Source: Front Plant Sci. 2026 Oct 14;17:1968539. doi: 10.3389/fpls.2026.1968539 (PMC13741887; doi:10.3389/fpls.2026.1968539)
Supplement: Supplementary file 1 [file Table1.docx]

**SUPPLEMENTARY INFORMATION FOR**

**Establishment of an efficient Agrobacterium rhizogenes-mediated hairy root transformation system for functional analysis in tomato**

Siyi Zhang^1^, Longzhu Huang^1^, Junjie Liu^1^, Zhimin Yin^1^, Ziwei Li^1^, Yuanhui Song^3^, Qiuling Zhang^1,2,^^[1]^*, Tengbo Huang^1,3,^^[2]^*

****TABLE S1 Antibiotics and concentrations used for selection of six Agrobacterium strains on Tryptone-Yeast extract (TY) plates/liquid medium.****

| *A. Rhizogenes* strain | Tryptone-Yeast extract (TY) of antibiotics | Tryptone-Yeast extract (TY) |
| --- | --- | --- |
| Ar Qual | 50mg/L streptomycin+50mg/L Kanamycin | The medium contained 5 g/L tryptone, 3 g/L yeast extract, and 1.11 g/L calcium chloride. For solid medium preparation, 15 g/L agar was additionally supplemented. |
| Ar A4 | 50mg/L Kanamycin |  |
| C58C1 | 100mg/L Rifampicin+50mg/L Kanamycin |  |
| K599 | 50mg/L streptomycin+50mg/L Kanamycin |  |
| MSU440 | 50mg/L streptomycin+50mg/L Kanamycin |  |
| Ar 1193 | 100mg/L Rifampicin+50mg/L Kanamycin |  |

****TABLE S2 Culture media for tomato hairy root transformation.****

| Culture medium | Component |
| --- | --- |
| MS basal medium | 4.74g/L MS basal salt mixture+30g/L sucrose+7g/L Gellan Gum |
| Co-cultivation medium | 4.74g/L MS basal salt mixture+30g/L sucrose+7g/L Gellan Gum+2mg/L Zeatin+0.5mg/L IAA+9.8mg/L Acetosyringone |
| hairy root induction MS medium | 4.74g/L MS basal salt mixture+30g/L sucrose+7g/L Gellan Gum+2mg/L Zeatin+0.5mg/L IAA+50mg/L Kanamycin+bacteriostatic antibiotics of TABLE 1 |

****TABLE S3 Minimum effective concentrations (MECs) of antibiotics determined by gradient screening for six Agrobacterium rhizogenes strains.****

| *A.rhizogenes* strain | 25mg/L Car | 50mg/L Car | 100mg/L Car | 200mg/L Car | 300mg/L Car | 400mg/L Car | 500mg/L Car | 600mg/L Car | 700mg/L Car | 800mg/L Car | 100mg/L Tim | 200mg/L Tim | 300mg/L Tim |
| --- | --- | --- | --- | --- | --- | --- | --- | --- | --- | --- | --- | --- | --- |
| ArQual | × | × | × | × | √ | √ | -- | -- | -- | -- | -- | -- | -- |
| ArA4 | × | × | × | √ | √ | √ | -- | -- | -- | -- | -- | -- | -- |
| C58C1 | -- | -- | -- | -- | × | × | × | √ | √ | -- | -- | -- | -- |
| MSU440 | -- | -- | -- | -- | × | × | × | × | × | × | √ | √ | √ |
| Ar1193 | -- | -- | -- | -- | × | × | × | × | × | × | √ | √ | √ |
| K599 | -- | -- | -- | -- | -- | -- | -- | × | × | × | √ | √ | √ |

The symbols used in the table are defined as follows: "√" represents antibiotic concentrations that effectively inhibited *A. rhizogenes* growth; "×" represents concentrations that did not inhibit bacterial growth; and "--" represents concentrations that were not included in the experiment.

****Table S4 Primer sequence****

| Primer name | Primer sequence(5′ → 3′) |
| --- | --- |
| *rolA*-F | GTTGCCTAGCGTTCGGACAGCCACCAGAG |
| *rolA*-R | GGCGTGGAAATGAATCGAAGACCGCCAGC |
| *rolB*-F | GCTCTTGCAGTGCTAGATTT |
| *rolB*-R | GAAGGTGCAAGCTACCTCTC |
| *rolC*-F | CTCCTGACATCAAACTCGTC |
| *rolC*-R | TGCTTCGAGTTATGGGTACA |
| *RUBY*-F | GCATCGAGAACACCGATAAGCTCACACTCC |
| *RUBY*-R | GGCAGCCACTCTGGGCGCATCTCTTCGTTG |
| *SlMYB28*-F | ATGAACAGTACATCTATGTCTTCTTTGGG |
| *SlMYB28*-R | CCAACCATCACTTTGTTCTTGTTGC |
| qRT-*SlActin*-F | TGTCCCTATCTACGAGGGTTATGC |
| qRT-*SlActin*-R | AGTTAAATCACGACCAGCAAGAT |
| qRT-*SlMYB28*-F | GCATCGATAGACAACGGAGATGAAT |
| qRT-*SlMYB28*-R | CCATAATCAGTTACACCTCCTCCTCC |

1. * Corresponding author.

   E-mail address: tengbohuang@szu.edu.cn
